# Haploinsufficiency of the *DMRT* Gene Cluster in a Case with 46,XY Ovotesticular Disorder of Sexual Development

**DOI:** 10.4274/balkanmedj.2017.0378

**Published:** 2018-05-29

**Authors:** Metin Eser, Akif Ayaz

**Affiliations:** 1 Clinic of Medical Genetics, Aydın State Hospital, Aydın, Turkey; 2 Clinic of Medical Genetics, University of Health Sciences, Kanuni Sultan Süleyman Training and Research Hospital, İstanbul, Turkey

**Keywords:** Genetic hybridization, haploinsufficiency, ovotesticular disorder of sex development

## Abstract

**Background:**

Ovotesticular disorder is characterized by the presence of testicular and ovarian tissues in the same individual. Single gene mutations in *SRY, SOX9, DMRT1* and *DAX1* can lead to ovotesticular disorder of sexual development.

**Case Report:**

Herein, we report a 3-month-old phenotypically female baby in whom differentiated tissues of both Müllerian and Wolffian ducts were detected on pathological analysis of laparoscopic biopsy material. Chromosomal analysis observed 46,XY, der(9)t(3;9)(p25;p24) with deletion of 9p24.3p23 including the DMRT gene cluster and duplication of 3p26.3p24.3 on array comparative genomic hybridisation.

**Conclusion:**

In support of previous literature, we found that haploinsufficiency of the *DMRT* gene cluster leads to ovotesticular disorder of sexual development. In addition, we emphasize that array comparative genomic hybridisation is an important technique in the molecular diagnosis of ovotesticular disorder of sexual.

Ovotesticular disorder is an extremely rare cause of disorder of sexual development (DSD), once known as true hermaphroditism. Patients with ovotesticular DSD have both testicular tissue, including seminiferous tubules, and ovarian tissue, including ovarian follicles. The gonads may be any combination of ovary, testes or combined ovary and testes called ovotestes. Although the external genitalia are usually ambiguous, they may vary from normal male to normal female. Ovotesticular disorder accounts for 3%-10% of all DSD patients. When the karyotypes of these patients are examined, 60% of them have 46,XX, 30% have 46,XX/46,XY and 10% have the 46,XY karyotype (1,2). Mosaicism, gene mutations and chimerism have been shown as primary causes of development of ovotesticular DSD. While development of dual gonads in the presence of 46,XX/46,XY or 46,XX/46,XXY may be easily explained, in the presence of 46,XX or 46,XY, the cause is more difficult to clarify, particularly if no *SRY* mutation is found. To date, mutations in the *SRY*, *SOX9* and *DMRT1* genes have been reported in only a few patients with 46,XY ovotesticular DSD (3,4,5). Herein, we report a case with the ovotesticular phenotype in whom was detected a 9p24.3-p23 deletion including the *DMRT* gene cluster region coincident with a 3p26.3-p24.3 duplication.

## CASE PRESENTATION

**Figure 1:**
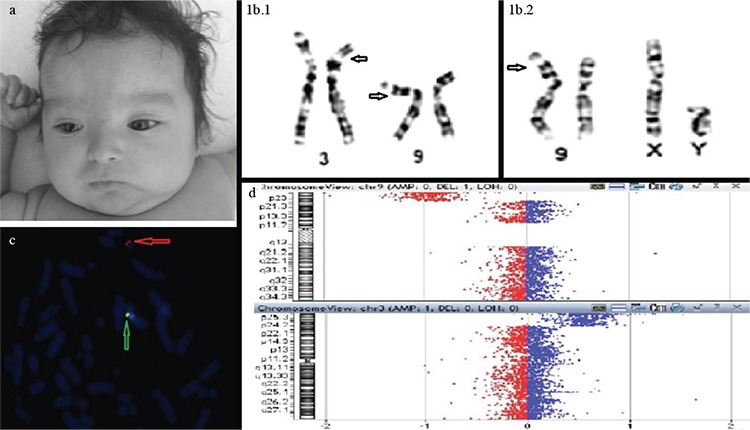
Images of the patient in front view. Capillary haemangiomas (forehead); hypertelorism; bilateral epicanthic folds; small nose and mouth; full cheeks; micrognathia and short, broad neck (a). Partial karyotype of the patient’s mother, t(3;9)(p25;p24) (1b.1). Partial karyotype of the patient, XY, der(9)t(3;9)(p25;p24). Arrows on the chromosomes indicate the translocation breakpoints (1b.2). Fluorescent *in situ* hybridization on metaphase chromosomes of the case with the LSI SRY(orange)/CEP X (green) probes. Metaphase spread showing a normal X chromosome (green signal for centromeric DXZ1 locus) and the presence of the SRY region (SRY orange) (c). Array-comparative genomic hybridisation profile of the patient showing the whole chromosome 9 (left) and an enlargement of the short arm with the 11.3 Mb deletion at 9p24.3-p23 (upper) and whole chromosome 3 (down) and an enlargement of the short arm with the 23.6 Mb duplication at 3p26.3-p24.3 (right) (d).

A 3-month-old female patient was referred to our Genetic Diagnosis Centre because of a dysmorphic appearance. The proband was born at full term to a 22-year-old female. She is the second-born child of a marriage between second cousins gravidity and parity. Birth weight was 3300 g (50^th^ centile); birth height and head circumference were unknown. She was hospitalized in an intensive care unit due to pneumonia during the neonatal period. Hypoglycaemia was present in her neonatal records. At her physical examination at 3 months of age, her height, weight and occipital-frontal circumference were 57.5 cm (25^th^-50^th^ centile), 6500 g (75^th^-90^th^ centile) and 40.3 cm (50^th^-75^th^ centile), respectively. She had a flat occiput, high forehead, capillary haemangiomas (forehead), hypertelorism, upward slanting palpebral fissure, bilateral epicanthic folds, posteriorly angulated ears, small nose and mouth, long philtrum, full cheeks, micrognathia, short and broad neck, widely spaced nipples and severe hypotonia (Figure 1a). On her external genitalia examination, mildly hypoplastic labia majora and prominent labia minora were observed. Ovaries were unclear on pelvic ultrasonography. Pelvic magnetic resonance imaging (MRI) findings indicated a small-sized uterus and a pericecal cystic lesion, while the adnexal structure imaging was unclear. On pathological analysis of laparoscopic paraovarian and paratubal cyst biopsy material, tissues differentiated as both Müllerian and Wolffian ducts were observed. While ovarian structure was observed on the right side, testis tissue with a spermatic cord was observed on the left side. Abdominal ultrasound revealed multiple cystic lesions and mild pelviectasis on the kidney. Mild hyperintensity on the frontal and parietal lobes was observed on cranial MRI. Echocardiogram showed mild patent foramen ovale. There were no specific finding on trans-fontanel ultrasound and electroencephalography. Written informed consent to the publication of the patient’s images was given by her parents. Chromosomal analysis of the proband and her relatives with their consent (father, mother) was carried out on cultured peripheral blood lymphocytes by using the standard protocol. In the next step, *SRY* fluorescence in situ hybridization (FISH) and array comparative genomic hybridisation (CGH) were planned. FISH was performed on metaphase chromosomes using an *SRY* probe (Cytocell, Banbury, UK) according to the manufacturer’s instructions. Array CGH was carried out on genomic DNA with an Agilent oligonucleotide array (Human Genome CGH microarray 4x180K; Agilent Technologies, Santa Clara, CA) according to the manufacturer’s instructions (probe alignments referred to the human February 2009, human genome 19 genomic assembly). A balanced reciprocal translocation, 46,XX, t(3;9)(p25;p24), was observed in her mother (Figure 1b.1). Chromosomal analysis of the proband revealed a 46,XY, der(9)t(3;9)(p25;p24) karyotype (Figure 1b.2). The father’s karyotype was normal. FISH results confirmed the presence of the *SRY* gene (Figure 1c). 

An 11.3 Mb distal deletion of 9p and a 23.6 Mb duplication of 3p, described as arr 9p24.3p23 (172,364-11,496,896)x1, 3p26.3p24.3 (117,735-23,802,377)x3, were shown by array CGH (Figure 1d).

## DISCUSSION

Detection of both ovarian and testicular tissues present in the same patient is called ovotesticular DSD. While the most common reported karyotypes are 46,XX or 46,XX/46,XY, 46,XY is extremely rare, occurring in approximately 10%-12.5% of ovotesticular DSD cases (2,6). At the gene level, mutations in the *SRY*, *SOX9*, *DMRT1* and *DAX1* genes have been reported as causes of ovotesticular DSD (3,4,5,7). In the current case, who is phenotypically female, 46,XY, der(9)t(3;9)(p25;p24) was detected in cultured peripheral blood lymphocytes. Due to the presence of both Müllerian and Wolffian ducts on laparoscopic paraovarian and paratubal cyst biopsy, the current case was evaluated as 46,XY ovotesticular DSD. In further genetic analysis, distal deletion of the 9p, including *DMRT* genes, along with duplication of the 3p were shown by array CGH. The short arm of chromosome 9, including the *DMRT* gene cluster, has been reported as one of the chromosomal regions related to DSD (8). *DMRT* proteins acting as transcription factors bind to the promoter regions of target genes via the zinc-finger domain manager domain. This DNA binding site is highly conserved. Target recognition of the consensus palindromic sequence of the DNA binding site requires protein dimerization (9). Raymond et al. (10) reported that *DMRT1* was expressed in the embryonic gonads of both sexes and in the foetal and adult mouse testis and was necessary for the postnatal differentiation of both somatic and germ cells in the testis. Another gene in the *DMRT* cluster, *DMRT2* is expressed in the adult testis. 

Chromosome 9p deletion syndrome (OMIM#158170) is a well-known disorder, a structural monosomy occurring with loss of a part of 9p of varying size. The most common findings of 9p deletion syndrome include developmental and psychomotor delay, dysmorphic features and developmental sex disorders in XY patients. In the present case, some findings, especially disorders of sex development, hypertelorism, small nose and mouth, long philtrum, micrognathia, widely spaced nipples and severe hypotonia, were compatible with 9p deletion syndrome. The common findings observed in partial duplication of 3p include mental and psychomotor retardation, microcephaly, short stature, short neck, gastrointestinal malformations, congenital heart defects, early postnatal death, hypoplastic genitalia, dysmorphic face such as frontal bossing, temporal indentation, hypertelorism and/or telecanthus, full cheeks and cleft lip/palate (11,12). Our case had some of these related anomalies, especially dysmorphic features. In patients having 9p deletion and 3p duplication, however, it is difficult to determine to what extent the dysmorphic stigmata are based upon each of the 3p duplication and the 9p deletion. We summarize the comparison of the clinical findings of 9p deletion syndrome and 3p duplication syndrome with those of our case in Table 1.

In conclusion, we report a case with contralateral 46,XY ovotesticular DSD, which is extremely rare, in whom was detected deletion of the *DMRT* gene cluster that is responsible for DSD. It should not be forgotten that mutations in *DMRT* genes as well as in *SRY*, *SOX9* and *DAX1* may lead to 46,XY ovotesticular DSD. In addition, array CGH is an important technique in the molecular diagnosis of ovotesticular DSD.

## Figures and Tables

**Table 1:**
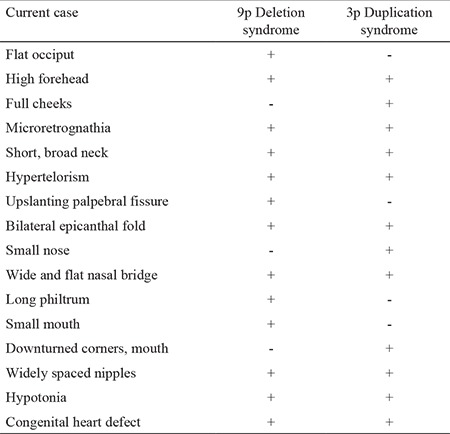
Comparison of the phenotypic manifestations in the current case with those of 9p deletion syndrome and 3p duplication syndrome

## References

[1] Hughes IA, Houk C, Ahmed SF, Lee PA, Lawson Wilkins Pediatric Endocrine Society/European Society for Paediatric Endocrinology Consensus Group (2006). Consensus statement on management of intersex disorders. J Pediatr Urol.

[2] Achermann JC, Hughes IA (Saunders: Philadelphia). Disorders of sex development. In: Melmed S, Polonsky KS, Larsen PR, Kronenberg HM, editors. William’s Textbook of Endocrinology, 12th ed.

[3] Maier EM, Leitner C, Löhrs U, Kuhnle U (2003). True hermaphroditism in an XY individual due to a familial point mutation of the SRY gene. J Pediatr Endocrinol Metab.

[4] Cameron FJ, Hageman RM, Cooke-Yarborough C, Kwok C, Goodwin LL, Sillence DO, et al (1996). A novel germ line mutation in SOX9 causes familial campomelic dysplasia and sex reversal. Hum Mol Genet.

[5] Ledig S, Hiort O, Wünsch L, Wieacker P (2012). Partial deletion of DMRT1 causes 46,XY ovotesticular disorder of sexual development. Eur J Endocrinol.

[6] Matsui F, Shimada K, Matsumoto F, Itesako T, Nara K, Ida S, et al (2011). Long-term outcome of ovotesticular disorder of sex development: a single center experience. Int J Urol.

[7] Ludbrook LM, Bernard P, Bagheri-Fam S, Ryan J, Sekido R, Wilhelm D, et al (2012). Excess DAX1 leads to XY ovotesticular disorder of sex development (DSD) in mice by inhibiting steroidogenic factor-1 (SF1) activation of the testis enhancer of SRY-box-9 (SOX9). Endocrinology.

[8] Tannour-Louet M, Han S, Corbett ST, Louet JF, Yatsenko S, Meyers L, et al (2010). Identification of de novo copy number variants associated with human disorders of sexual development. PLoS One.

[9] Erdman SE, Chen HJ, Burtis KC (1996). Functional and genetic characterization of the oligomerization and DNA binding properties of the Drosophila doublesex proteins. Genetics.

[10] Raymond CS, Murphy MW, O’Sullivan MG, Bardwell VJ, Zarkower D (2000). DMRT1, a gene related to worm and fly sexual regulators, is required for mammalian testis differentiation. Genes Dev.

[11] Conte RA, Pitter JH, Verma RS (1995). Molecular characterization of trisomic segment 3p24.1-->3pter: a case with review of the literature. Clin Genet.

[12] Reiss JA, Sheffield LJ, Sutherland GR (1986). Partial trisomy 3p syndrome. Clin Genet.

